# Elective and emergency laparoscopic cholecystectomy in the elderly: our experience

**DOI:** 10.1186/1471-2482-13-S2-S21

**Published:** 2013-10-08

**Authors:** Alessia G Ferrarese, Mario Solej, Stefano Enrico, Alessandro Falcone, Silvia Catalano, Giada Pozzi, Silvia Marola, Valter Martino

**Affiliations:** 1 University of Turin, Department of Oncology, School of Medicine, Teaching Hospital "San Luigi Gonzaga", Section of General Surgery, Orbassano, Turin, Italy

## Abstract

**Background:**

We aimed to analyze outcomes of early and delayed laparoscopic cholecystectomy in the elderly in our General Surgery Division.

**Methods:**

We analyzed 114 LC performed from the 1st of January 2008 to the 31st of December 2012 in our General Surgery division: 67 LC were performed for gallbladder stones and 47 for acute cholecystitis.

**Results and discussion:**

Comparison between Ordinary and Emergency groups showed that drain placement and post-operative hospital stay were significatively different. There were no significative differences between Early Laparoscopic Emergency Cholecystectomy (E-ELC) and Delayed Laparoscopic Emergency Cholecystectomy (D-ELC). There weren't any differences about Team's evaluation.

**Conclusion:**

We consider LC a safe and effective treatment for cholelitiasis and acute cholecystitis in Ordinary and Emergency setting, also in the elderly. We also demonstrate that, in our experience, LC for AC is feasible as well.

## Background

Laparoscopic cholecystectomy (LC) represents the gold standard treatment for cholelithiasis.

Its application gradually extended to acute cholecystitis (AC) also in the elderly. We aimed to compare outcomes of the University Section of General Surgery in "San Luigi Gonzaga" Hospital of Orbassano (Turin) with literature, evaluating timing and technique of early or delayed laparoscopic cholecystectomy in the management of acute cholecystitis in elderly patients.

## Methods

From the 1st of January 2008 to the 31st of December 2012, 114 LC were performed at the University Section of General Surgery in elderly patients (Age > 65 yrs): 67 for gallbladder stones and 47 for acute cholecystitis. The diagnosis of cholecystitis and gallbladder stones was performed basing on general conditions, physical examination, laboratory exams, radiologic findings and sepsis score. For the study we also considered: total hospital stay, time before and after surgery, duration and kind of operation, conversion to open procedure, drain and final pathological results. We excluded 29 patients from the study (17 for choledocolytiasis associated and 12 for hospitalisation > 20 days). We didn't exclude ASA III and ASA IV patients: in these patients (27,4%, 17 ASA III and 4 ASA IV) we used abdominal pressure not superior of 10 mmHg [1]. We included in the study 85 elderly patients (49 M, 36 F): Ordinary Cholecystectomy was peformed in 45 cases (Ordinary Group) and Emergency Cholecystectomy in 40 cases (Emergency Group). This last group was further divided in two groups [2-4]: E-ELC (31 patients with surgery performed before 72 hours from starting of the symptoms) and D-ELC, (9 patients with surgery performed after 72 hours until 9 day). The experience of the first operator was also considered a contributing factor. Basing on this factor, and considering laparoscopic learning curves as described in literature (29-40), we identified three subgroups of surgery teams (Table 1) in order to evaluate our results [5-11].

**Table 1:** Definitions of team according to the experience of the lead surgeon

Team 1	More than 100 laparoscopic cholecistectomy and more than 100 other laparoscopic operations.
Team 2	Less than 100 laparoscopic cholecistectomy and less than 100 other laparoscopic operations.

Team 3	Surgeons in learning curve progression or Resident with expert Surgeon supervisor

Statistical proportions related to the analyzed dichotomic variables, for both E-ELC and D-ELC (gender distribution in different patient groups, number of post-operative complications, conversion rate, number of drains, number of other related surgeries, presence of fever, wall thickening, effusion amount, gallbladder distension and calculosis type) were compared using Chi-square test and Fisher's exact test. Continuous variables like age distribution, post-operative hospital stay time, surgery duration and several haematochemical characteristics (WBC, CRP) were expressed as average (range) and analyzed using the Mann-Witney U test. Patient distribution according to different surgical teams was confirmed. All statistical analyses were performed using R software (*version 2.6.2*), and a *p *value of less than 0.01 was considered indicative of statistical significance.

## Results and discussion

In our experience, the comparison between Ordinary and Emergency Group was no statistically significant about blood test values and ultrasonographic evidence (Table 2).

**Table 2:** Statistical analysis based on the comparison of Ordinary vs DEA Groups

	Ordinary Group	Emergency Group	P Value
Operation time (min)	75,5 (40-220)	90 (28-200)	0,1874

PO hospital stay (days)	2 (1-10)	3 (2-12)	0,002313

Conversion rate	6,7%	2%	0,3869

Complications	8,5%	2%	0,2352

Drains	16,7%	51%	0,0003

Associated operations	13,3%	12,8%	0,998

Cancer	3%	0	-

We analyzed E-ELC and D-ELC data without finding any statistically significant difference in the elderly, except for the full hospital stay duration, which was longer for D-ELC patients (Table 3). Operation time, conversion rate, and complications did not demonstrate any significant difference between the two groups. Comparison of success rates achieved by different surgeons yielded the same results, regardless of their levels of experience (Table 4). Patients can be operated after a time interval of 73 hours and up to 9 days, and receive the same benefits that would have been obtained from an earlier operation.

**Table 3:** Statistical analysis based on the comparison of E-DLC and D-DLC Groups

	Early-ELC	Delayed-ELC	P Value
WBC	11,05 (3,73-28,8)	9,05 (2,23-15,6)	0,03264

PCR	1,39 (0,04-45)	0,66 (0,08-23,23)	0,1672

Temperature	14%	2 (7%)	0,5281

Thickened wall	57.4%	13 (48%)	0,4

Pericholecystic fluid	17%	2 (7.4%)	0,25

Distended gallbladder	43.4%	12 (44.4%)	0,998

Operation time (min)	90 (36-330)	85 (28-195)	0,1554

PO hospital stay (days)	3 (2-15)	3 (2-8)	0,6551

Total hospital stay	4 (2-16)	10 (4-16)	p < 0,01

Tasso di conversione	5%	0%	0,59

Complications	5%	0%	0,59

Drains	36%	26%	0,3752

Operations associated	8%	15%	0,2353

Cancer	1,6%	0%	0,998

**Table 4:** Statistical analysis based on the Team

	Team 1-Team 2	Team 1-Team 3	Team 2-Team 3
Operation time (min)	0,6936	0,6089	0,2759

PO hospital stay (days)	0,3159	0,02131	0,09583

Total hospital stay	0,9362	0,004337	0,004981

Conversion rate	0,1553	0,6677	0,3896

Complications	0,3823	0,998	0,998

## Conclusions

In agreement with literature [8-10], we consider LC a safe and effective treatment for AC also in the elderly. This study demonstrates that in our experience LC for AC is feasible as well. The learning curve of this procedure is feasible [11,12]. We also believe that, whenever possible, early LC is to be preferred, above all for the significantly shortened total hospital stay. Nevertheless, the retrospective analysis of our case study, even with a smaller sample for delayed LC patients, showed that elderly patients can be operated with delayed approach and still benefit from the same advantages that would be obtained with an early operation [12-19]. In our experience, according to literature, laparoscopic cholecystectomy is a secure procedure to be performed [20-24]. We consider surgery approach more difficult in the elderly in some cases [25] but we also considered laparoscopic approach is, in general, a safe and feasible technique in acute pathology and a safe approach also in the elderly [26].

## Competing interests

The authors declare that they have no competing interests.

## Authors' contributions

AGF: conception and design, interpretration of data, given final approval of the version to be published.

SE: conception and design, interpretration of data, given final approval of the version to be published.

MS: acquisition of data, drafting the manuscript, given final approval of the version to be published.

AF: acquisition of data, drafting the manuscript, given final approval of the version to be published.

SC: acquisition of data, drafting the manuscript, given the final approval of the version to be published.

GP: acquisition of data, drafting the manuscript, given the final approval of the version to be published.

SM: critical revision, interpretation of data, given final approval of the version to be published

VM: critical revision, interpretation of data, given final approval of the version to be published

## Declarations

Funding for this article came from personal funds.

This article has been published as part of *BMC Surgery *Volume 13 Supplement 2, 2013: Proceedings from the 26th National Congress of the Italian Society of Geriatric Surgery. The full contents of the supplement are available online at http://www.biomedcentral.com/bmcsurg/supplements/13/S2

## References

[B1] Catani M Modini C Laparoscopic cholecystectomy in acute cholecystitis: a proposal of safe and effective technique Hepatogastroenterology 2007 13 2186 91 18265630

[B2] Chung-Mau Lo Chi-Leung Liu Sheung-Tat Fan Edward Lai CS John Wong Prospective Randomized Study of Early Versus Delayed Laparoscopic Cholecystectomy for Acute Cholecystitis Ann Surg 2008 13 461 467 10.1097/00000658-199804000-00001 PMC1191296 9563529

[B3] Litwin DE Cahan MA Laparoscopic cholecystectomy Surg Clin North Am 2008 13 1295 313 18992596 10.1016/j.suc.2008.07.005

[B4] Wilson E Gurusamy K Gluud C Davidson BR Cost-utility and value-of-information analysis of early versus delayed laparoscopic cholecystectomy for acute cholecystitis Br J Surg 2010 13 210 219 20035545 10.1002/bjs.6872

[B5] Dàvila D Manzanares C Picho ML Albors P Cardenas F Fuster E Experience in the treatment (early vs. delayed) of acute cholecystitis via laparoscopy Cirugia Espanola 1999 13 233 235

[B6] Bohacek L David MD Pace E Advanced laparoscopic training and outcomes in laparoscopic cholecystectomy Can J Surg 2009 13 223 224 PMC2724801 19680513

[B7] Ballantyne GH Ewing D Capella RF The learning curve measured by operating times for laparoscopic and open gastric bypass: roles of surgeon's experience, institutional experience, body mass index and fellowship training Obes Surg 2005 13 172 82 15810124 10.1381/0960892053268507

[B8] Gill J Booth MI Stratford J Dehn TC The extended learning curve for laparoscopic fundoplication: a cohort analysis of 400 consecutive cases J Gastrointest Surg 2007 13 487 92 17436134 10.1007/s11605-007-0132-0 PMC1852390

[B9] Avital S Hermon H Greenberg R Karin E Skornick Y Learning curve in laparoscopic colorectal surgery: our first 100 patients Isr Med Assoc J 2006 8 683 6 17125113

[B10] Rispoli C Rocco N Iannone L Amato B Developing guidelines in geriatric surgery: role of the grade system BMC Geriatrics 2009 13 SUPPL.1 A99

[B11] Li GX Yan HT Yu J Lei ST Xue Q Cheng X Learning curve of laparoscopic resection for rectal cancer 2006 13 535 8 16624777

[B12] Kauvar DS Brown BD Braswell AW Harnisch MJ Laparoscopic cholecystectomy in the elderly: increased operative complications and conversions to laparotomy Laparoendosc Adv Surg Tech A 2005 13 379 82 10.1089/lap.2005.15.379 16108740

[B13] Moyson J Thill V Simoens Ch Smets D Debergh N Mendes da Costa P Laparoscopic cholecystectomy for acute cholecystitis in the elderly: a retrospective study of 100 patients Hepatogastroenterology 2008 13 1975 80 19260462

[B14] Polychronidis A Botaitis S Tsaroucha A Tripsianis G Bounovas A Pitiakoudis M Simopoulos C Laparoscopic cholecystectomy in elderly patients J Gastrointestin Liver Dis 2008 17 309 13 18836625

[B15] Kirshtein B Bayme M Bolotin A Mizrahi S Lantsberg L Laparoscopic cholecystectomy for acute cholecystitis in the elderly: is it safe? Surg Laparosc Endosc Percutan Tech 2008 13 334 9 18716529 10.1097/SLE.0b013e318171525d

[B16] Majeski J Laparoscopic cholecystectomy in geriatric patients Am J Surg 2004 13 747 50 15191870 10.1016/j.amjsurg.2003.11.031

[B17] Stanisić V Bakić M Magdelinić M Kolasinac H Miladinović M Laparoscopic cholecystectomy in elderly patients Acta Chir Iugosl 2009 13 87 91 19780336 10.2298/aci0902087s

[B18] Tambyraja AL Kumar S Nixon SJ Outcome of laparoscopic cholecystectomy in patients 80 years and older World J Surg 2004 13 745 8 15457351 10.1007/s00268-004-7378-4

[B19] Aprea G Canfora A Ferronetti A Giugliano A Guida F Braun A Battaglini Ciciriello M Tovecci F Mastrobuoni G Cardin F Amato B Morpho-functional gastric pre-and post-operative changes in elderly patients undergoing laparoscopic cholecystectomy for gallstone related disease BMC Surgery 2012 13 Suppl 1 S1 23173777 10.1186/1471-2482-12-S1-S5 PMC3499270

[B20] Lau H Lo CY Patil NG Yuen WK Early versus delayed-interval laparoscopic cholecystectomy for acute cholecystitis: a metaanalysis Surg Endosc 2006 13 82 7 16247580 10.1007/s00464-005-0100-2

[B21] Johansson M Thune A Blomqvist A Nelvin L Lundell L Management of acute cholecystitis in the laparoscopic era: results of a prospective, randomized clinical trial J Gastrointest Surg 2003 13 642 645 12850677 10.1016/s1091-255x(03)00065-9

[B22] Lo C Liu C Fan ST Lai EC Wong J Prospective randomized study of early versus delayed laparoscopic cholecystectomy for acute cholecystitis Ann Surg 1998 13 461 467 9563529 10.1097/00000658-199804000-00001 PMC1191296

[B23] Solej M Martino V Mao P Enrico S Rosa R Fornari M Destefano I Ferrarese AG Gibin E Bindi F Falcone A Ala U Nano M Early versus delayed laparoscopic cholecystectomy for acute cholecystitis Minerva Chirurgica 2012 13 5 381 387 23232475

[B24] Ferrarese A Martino V Nano M Elective and emergency laparoscopic cholecystectomy in the elderly: early or delayed approach BMC Geriatrics 2011 13 Suppl 1 A14 10.1186/1471-2482-13-S2-S21 PMC3851193 24268106

[B25] Ferrarese A Martino V Falcone A Solej M Destefano I Perforated duodenal diverticulum: case report and short review of the literature in press su Chirurgia

[B26] Ferrarese A Martino V Nano M Wound defects in the elderly: our experience BMC Geriatrics 2011 13 Suppl 1 A15 10.1186/1471-2482-13-S2-S23 PMC3851270 24266927

